# An introduction to the special issue: The ecological approach of James J. Gibson: 40 years later

**DOI:** 10.1177/20416695221123865

**Published:** 2022-10-06

**Authors:** H. A. Sedgwick, Brian Rogers

This *i-Perception* Special Issue on “The Ecological Approach of James J. Gibson: 40 years later” is based on the ECVP2019 symposium of the same name. The aim of the symposium was to discuss and evaluate Gibson's legacy, 40 years after the publication of his last book *“The Ecological Approach to Visual Perception.”* The symposium speakers were Bill Warren, Jim Todd, Brian Rogers, Hal Sedgwick, Ken Nakayama, and Barbara Gillam. Five of the six speakers have written papers for this Special Issue, based on their presentations in the Symposium. A further four papers by Joe Lappin, Diederick Niehorster, Geoffrey Bingham, and John Kennedy were also accepted for publication. Hal Sedgwick submitted a final paper for the Special Issue in which he summarizes the contribution of the Arabic scholar, Ibn al-Haytham, who argued for the role of the ground plane in making judgements of distance and space perception—more than a thousand years before Gibson!

**Figure 1.**
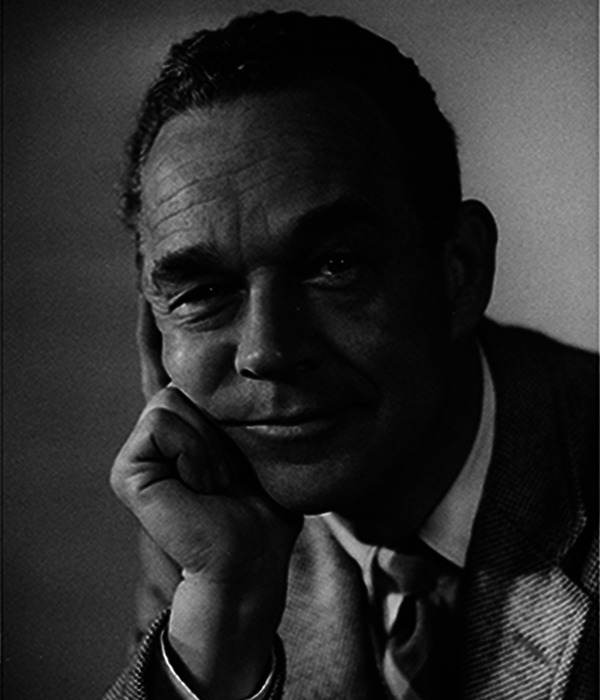
A photograph of James Gibson taken at the time (1955) he was awarded a Fullbright Fellowship to spend a year in Oxford.

## Gibson's Life and Career^1^

James Jerome Gibson, commonly referred to as “J. J.”, was born in 1904 in the American Midwest and grew up there. He attended Princeton University, graduating in 1925, and then staying on to study the behaviorist approach to learning. After completing his PhD there in 1928, Gibson was hired by Smith College, an undergraduate college for women, where he stayed for 14 years. This was an important time for Gibson in several ways. In 1932, he married Eleanor Jack, a former student of his. Eleanor Jack Gibson was determined to have a career in research herself, and she went on to do graduate work at Yale with Clark Hull, a leading theorist of learning. She received her PhD in 1938.

During the 1930s, J. J. Gibson was establishing a reputation based on his research in perception, particularly his discovery of the adaptation and aftereffects produced by prolonged exposure to figural forms such as tilt or curvature. Gibson's years at Smith also exposed him to Gestalt psychology. Kurt Koffka, one of the founders of Gestalt Psychology was at Smith. In 1935, Koffka published *“Principles of Gestalt Psychology,”* which made available a detailed exposition of Gestalt theory and research in English. That book, as well as his conversations with Koffka, deeply influenced Gibson's thinking. Gibson never lost sight of Koffka's importance and, many years later when one of us was studying with Gibson at Cornell, his research seminar devoted an entire semester to a careful reading and analysis of Koffka's *“Principles.”*

The Gestalt psychologist Fritz Heider and his wife were also at Smith, and they became personal friends of the Gibsons. It was Fritz Heider who introduced J. J. Gibson to the work of Kurt Lewin, and in particular, to his *“Principles of Topological Psychology,”* which appeared in 1936. The book inspired Gibson's article: “A Theoretical Field-Analysis of Automobile-Driving,” published with Laurence Crooks in 1938. This may have been Gibson's first effort to analyze the information used to control visually-guided action.

Following the entry of the U.S. into WWII, Gibson was recruited into the war effort. There was an urgent need to rapidly increase the number of airplane pilots in the military, but little understanding of how to select and to train them. The task of improving this effort was assigned to a group led by Gibson. They did research on a wide range of related perceptual problems throughout the war years. It was in struggling with these very practical problems that Gibson gradually developed a new theoretic approach to visual perception.

**Figure 2.**
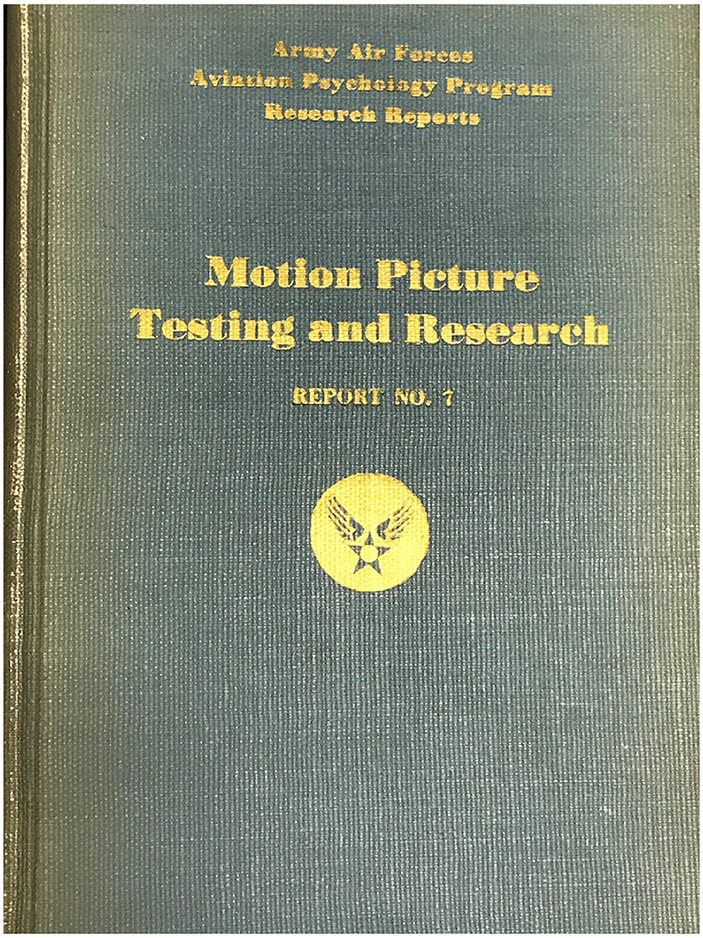
The cover of Gibson's final report to the US Army in 1947.

Many of his new ideas appeared in print for the first time in 1947 in Gibson's final report to the army, summarizing his group's accomplishments.

Following WWII, Gibson returned briefly to Smith, but in 1949 he was recruited to join the Department of Psychology at Cornell University, where he stayed for the remainder of his career.

During this time, Eleanor Gibson, often referred to as “Jackie,” was continuing to pursue her own career. The hurdles that all women in academia faced at that time were only raised higher by being married to a male academic. Although Jackie Gibson already had a formidable body of published research by the time J. J. Gibson was offered a position at Cornell, there were rules against “nepotism,” which seemed to be aimed entirely at the wives of male faculty, and this prevented her from receiving any faculty position at Cornell. Only in 1966, when J. J. received a grant that allowed him to retire permanently from teaching, did Cornell give Jackie Gibson a faculty position, which she then occupied with great distinction for several decades more.

After a few successful, but perhaps also stressful, collaborations, the Gibsons decided to go their separate ways in their research efforts, splitting the field of visual perception between them, with Jackie concentrating on learning and development. They had separate labs and each had their own students, but students often attended seminars with both of them, and they continued to share and to develop a common theoretical perspective.

In 1950, J. J. Gibson brought out *“The Perception of the Visual Field and the Visual World,”* in which he developed his ideas into a coherent and comprehensive theoretical approach.

**Figure 3.**
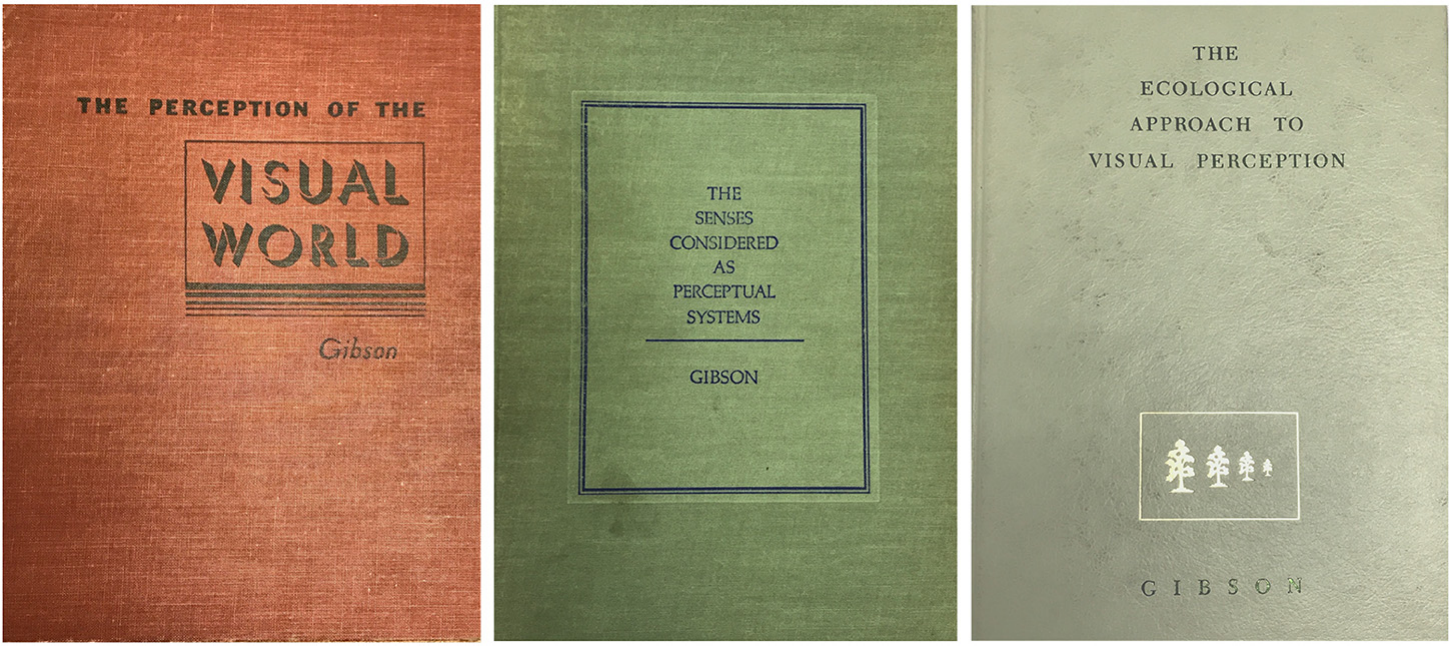
The covers of J. J. Gibson's three books: *The Perception of the Visual World* (1950); *The Senses Considered as Perceptual Systems* (1966); and *The Ecological Approach to Visual Perception* (1979).

In 1966, *“The Senses Considered as Perceptual Systems”* was published, in which Gibson extended his theoretical framework beyond vision to take in all of the perception.

And finally in 1979, he returned to vision in *“The Ecological Approach to Visual Perception,”* which presents the theoretical developments produced by 30 years of thinking and of research by Gibson and his students, as well as other new work that he saw as related.

J. J. Gibson died of pancreatic cancer in December of the same year, 1979.

## The Purpose and Objectives of the Symposium and the Special Issue

The principal objective of both the symposium and this Special Issue was to evaluate the legacy of the ideas contained in Gibson's 1979 book. This necessarily implies an evaluation of the idea of “direct” perception and why he rejected the role of so-called higher-level processes of “inference” (Hermann von Helmholtz), “perceptual hypotheses” (Richard Gregory), “intelligent, thought-like processes” (Irv Rock). In addition, Gibson rejected the traditional assumption of insufficiency of the information reaching our senses in favor of the claim that there is a richness of information in the natural, ecological world in which we have evolved and now live. As a consequence, our aim has been to provide answers to the question of whether, and how, these ideas have affected the way we study perception in the 40 years since the publication of *“The Ecological Approach to Visual Perception.”*

It is worth remembering that there have been many changes over the last 40 years in both the technologies that we use—from oscilloscopes to large-field displays, virtual reality and head-mounted devices—and in the characteristics of the *stimuli* we present to observers—from spatial frequency gratings, random dot patterns and simple line drawings to real-world images and changing optic flow sequences. At the same time, there have been huge changes in the models we use to test and complement our experimental findings, from David Marr's algorithmic approach in the 1980's to the more recent convolutional neural networks.

The major areas of research in perception and vision science have also changed. In the program of the 1979 ECVP meeting, presentations on spatial and temporal processing, grating patterns, VEPs, color vision, eye movements, and physiology dominated. The notable exception to predominance of papers and posters on low-level vision was Jan Koenderink's invited lecture entitled *“Light on solid shape”*—clearly, way ahead of its time! The psychophysics section of the ARVO meeting in 1980 was similarly dominated by papers and posters on low-level aspects of vision—rods, cones and adaptation, spatial and temporal channels, and color vision. There was just a single paper session labelled “Perception”—which, we assume, was intended to include all other aspects of vision research including illusions.

The topics of the sessions in this year's ECVP2022 meeting have little in common with those of 40 years ago. They include “Scene Perception,” “Perception and Action,” “Multi-sensory Perception,” “Optic Flow,” and “Surface and Texture.” How much of the research reported at this year's conference has been influenced by J. J. Gibson's ideas? An impossible question to answer and, even if the research was influenced by Gibson's ideas, it is not always appreciated or acknowledged. However, it is clear that Gibson's ideas about surfaces, optic flow, information rather than stimuli, the close link between perception and action, and the importance of studying perception in natural rather than impoverished environments, are now important themes of current research. Even Gibson's most controversial idea of “affordances”—thinking about perception as discovering the *meaning* of what the world offers—has crept into the way we think about perception.

As Editors of the Special Issue, we were delighted to receive all the submissions but, at the same time, we were a little disappointed that there were no submissions from those who disagree with Gibson's approach and ideas. It is now too late to incorporate such views in our Special Issue but we hope that the Editorial section of *Perception* might be an appropriate forum for anyone interested in presenting an alternative view.

## Notes

1. The biographical information presented here is drawn from several sources (Gibson, E.J., 2002; Gibson, J. J., 1967; Hochberg, J., 1994), as well as from the personal recollections of the authors.
